# Latest trends in cancer incidence among UK South Asians in Leicester

**DOI:** 10.1038/sj.bjc.6600973

**Published:** 2003-07-01

**Authors:** L K Smith, J L Botha, A Benghiat, W P Steward

**Affiliations:** 1 Department of Epidemiology and Public Health, University of Leicester, 22-28 Princess Road West, Leicester LE1 6TP, UK; 2 Trent Cancer Registry, Weston Park Hospital, Whitham Road, Sheffield S10 2SJ, UK; Department of Oncology, Leicester Royal Infirmary, Leicester LE1 5WW, UK; 3 Department of Oncology, Leicester Royal Infirmary, Leicester LE1 5WW, UK

**Keywords:** incidence, ethnicity

## Abstract

Using cancer registry data, we show that although South Asians have lower rates of cancer than the rest of the population, this is changing with age and time. Younger South Asians, particularly children, are at increased risk. While generally cancer rates have fallen over the last decade, they are increasing among South Asians.

Cancer incidence rates in the UK have been reported to be much lower for South Asians (Indian, Pakistani and Bangladeshi) than the rest of the population (Winter *et al*, 1999). However, there has been little report on recent trends in cancer among South Asians in the UK. In order to explore recent cancer incidence trends, we used 1990–1999 cancer registry data for Leicester, UK, a city where 22% of residents classified themselves as South Asian in the 1991 census.

## MATERIALS AND METHODS

Data were obtained from Trent Cancer Registry on all registrations of cancer diagnosed between 1 January 1990 and 31 December 1999, where the patient was a Leicester resident at diagnosis. Since routine ethnicity data were not available for all patients, individuals were classified as South Asian or non-South Asian based on forename and surname. Software was used to do this (Cummins *et al*, 1999), but accuracy was increased by visual inspection of the data. Deprivation is often a confounder in studies of ethnicity since they are closely related. There have been shown to be trends in incidence with deprivation for various cancer sites (Pollock and Vickers, 1997) so it was important to take account of deprivation in the analyses of cancer incidence. Therefore, the Townsend Index (Townsend *et al*, 1988) was used to assess the level of deprivation of the patient's area of residence. The local research ethics committee approved the study.

Data were analysed for all cancers combined (all ages) and then for five common cancer sites (patients age 30 years and over): colorectal, head and neck, breast, lung and prostate. Electoral ward level population estimates were obtained from the 1991 census (by sex, ethnicity and 5-year age bands) and aggregated by deprivation tertile in order to calculate incidence rates. Variation in incidence rates by ethnicity, deprivation tertile, age and year of diagnosis (and any interactions between them) was explored using Poisson regression, separately for men and women. Firstly, a model was fitted to estimate the incidence rate ratio (IRR) for South Asians compared with non-South Asians for each age group, adjusting for deprivation tertile, and an interaction between ethnicity and age group was fitted to explore differential trends with age (Table 1

**Table 1:** **Incidence rate ratios** (95% Confidence Intervals) comparing South Asians with non-South Asians by sex, cancer site and age, adjusted for deprivation tertile.

		**Age at diagnosis (years)**					
	**All ages**	**0–14**	**15–29**	**30–49**	**50–74**	**75+**	***χ*^2^ statistic (*P*-value) change in IRR for ethnicity by age**
**Males**							
All cancers	**0.61**	**2.56**	**1.18**	**0.68**	**0.55**	**0.59**	*χ*^2^_4_=26.4
	(0.55, 0.68)	(1.33, 4.93)	(0.73, 1.92)	(0.51, 0.90)	(0.48, 0.64)	(0.47, 0.74)	(*P*=0.0001)
	*N*_SA_=401, *N*_NSA_=5535	*N*_SA_=19, *N*_SA_=17	*N*_SA_=22, *N*_SA_=66	*N*_SA_=59, *N*_NSA_=276	*N*_SA_=223, *N*_NSA_=3097	*N*_SA_=78, *N*_NSA_=2079	
Colorectal	**0.44**			**0.94**	**0.50**	**0.10**	*χ*^2^_2_=10.96
	(0.32, 0.61)			(0.43, 2.08)	(0.34, 0.73)	(0.02, 0.40)	(*P*=0.0042)
	*N*_SA_=38, *N*_NSA_=778			*N*_SA_=8, *N*_NSA_=30	*N*_SA_=28, *N*_NSA_=431	*N*_SA_=2, *N*_NSA_=317	
Head and neck	**1.53**			**1.08**	**1.28**	**3.64**	*χ*^2^_2_=6.8
	(1.09, 2.15)			(0.42, 2.76)	(0.83, 1.96)	(1.87, 7.09)	(*P*=0.033)
	*N*_SA_=43, *N*_NSA_=197			*N*_SA_=6, *N*_NSA_=17	*N*_SA_=26, *N*_NSA_=136	*N*_SA_=11, *N*_NSA_=44	
Lung	**0.40**			**0.53**	**0.40**	**0.37**	*χ*^2^_2_=0.41
	(0.31, 0.53)			(0.21, 1.37)	(0.29, 0.55)	(0.20, 0.67)	(*P*=0.81)
	*N*_SA_=55, *N*_NSA_=1187			*N*_SA_=5, *N*_NSA_=32	*N*_SA_=39, *N*_NSA_=724	*N*_SA_=11, *N*_NSA_=431	
Prostate	**0.52**			-	**0.47**	**0.60**	*χ*^2^_1_=0.45
	(0.37, 0.72)				(0.31, 0.72)	(0.36, 1.00)	(*P*=0.50)
	*N*_SA_=38, *N*_NSA_=856			*N*_SA_=0, *N*_NSA_=2	*N*_SA_=23, *N*_NSA_=429	*N*_SA_=15, *N*_NSA_=425	
							
*Person-years*	PY_SA_=347 960	PY_SA_=107 300	PY_SA_=82 050	PY_SA_=105 820	PY_SA_=48 350	PY_SA_=4440	
	PY_NSA_=1206 950	PY_NSA_=245 190	PY_NSA_=287 120	PY_NSA_=299 330	PY_NSA_=304 850	PY_NSA_=70 460	
							
**Females**							
All cancers	**0.75**	**1.66**	**1.15**	**0.71**	**0.71**	**0.77**	*χ*^2^_4_=9.79
	(0.68, 0.82)	(0.89, 3.12)	(0.72, 1.83)	(0.58, 0.86)	(0.62, 0.81)	(0.60, 1.00)	(*P*=0.044)
	*N*_SA_=461, *N*_NSA_=5731	*N*_SA_=17, *N*_NSA_=23	*N*_SA_=24, *N*_NSA_=69	*N*_SA_=122, *N*_NSA_=567	*N*_SA_=238, *N*_NSA_=2741	*N*_SA_=60, *N*_NSA_=2331	
Breast	**0.61**			**0.45**	**0.71**	**0.65**	*χ*^2^_2_=4.79
	(0.50, 0.73)			(0.32, 0.63)	(0.56, 0.90)	(0.34, 1.27)	(*P*=0.091)
	*N*_SA_=121, *N*_NSA_=1477			*N*_SA_=36, *N*_NSA_=273	*N*_SA_=76, *N*_NSA_=768	*N*_SA_=9, *N*_NSA_=436	
Colorectal	**0.51**			**1.11**	**0.39**	**0.46**	*χ*^2^_2_=4.92
	(0.35, 0.75)			(0.54, 2.28)	(0.23, 0.68)	(0.21, 1.04)	(*P*=0.086)
	*N*_SA_=30, *N*_NSA_=776			*N*_SA_=10, *N*_NSA_=30	*N*_SA_=14, *N*_NSA_=333	*N*_SA_=6, *N*_NSA_=413	
Head and neck	**2.97**			**4.58**	**1.99**	**3.48**	*χ*^2^_2_=3.95
	(2.02, 4.37)			(2.46, 8.52)	(1.10, 3.60)	(1.23, 9.83)	(*P*=0.14)
	*N*_SA_=42, *N*_NSA_=121			*N*_SA_=24, *N*_NSA_=18	*N*_SA_=14, *N*_NSA_=64	*N*_SA_=4, *N*_NSA_=39	
Lung	**0.32**			-	**0.31**	**0.65**	*χ*^2^_1_=2.07
	(0.20, 0.50)			*N*_SA_=0, *N*_NSA_=30	(0.18, 0.53)	(0.29, 1.47)	(*P*=0.15)
	*N*_SA_=20, *N*_NSA_=637				*N*_SA_=14, *N*_NSA_=372	*N*_SA_=6, *N*_NSA_=235	
							
*Person-years*	PY_SA_=355 200	PY_SA_=104 230	PY_SA_=93 260	PY_SA_=104 580	PY_SA_=48 220	PY_SA_=4910	
	PY_NSA_=1311 820	PY_NSA_=231 940	PY_NSA_=303 080	PY_NSA_=301 120	PY_NSA_=335 280	PY_NSA_=140 400	

Number of cancer cases and Person-years for South Asians: *N*_SA,_ PY_SA_; Number of cancer cases and Person-years for non-South Asians *N*_NSA_, PY_NSA_.

). An interaction between time period (1990–1994 and 1995–1999) and ethnicity was then included to compare changes in incidence over time between South Asians and non-South Asians (Table 2

**Table 2:** **Incidence rate ratios** (95% confidence intervals) comparing incidence rates for 1995–1999 with 1990–1994, by ethnicity, sex and cancer site adjusted for age and deprivation tertile

	**Ethnicity**		
	**South Asians**	**Non-South Asians**	***χ*^2^ statistic (*P*-value) for change in IRR between time periods by ethnicity**
**Males**			
All cancers	**1.28**	**0.95**	*χ*^2^_1_=8.01 (*P*=0.0047)
	(1.05, 1.56)	(0.90, 1.00)	
	*N*_90–94_=176, *N*_95–99_=225	*N*_90–94_=2834, *N*_95–99_=2701	
Colorectal	**1.24**	**1.03**	*χ*^2^_1_=0.31 (*P*=0.58)
	(0.65, 2.34)	(0.89, 1.18)	
	*N*_90–94_=17, *N*_95–99_=21	*N*_90–94_=384, *N*_95–99_=394	
Head and neck	**0.95**	**0.88**	*χ*^2^_1_=0.06 (*P*=0.80)
	(0.52, 1.74)	(0.66, 1.16)	
	*N*_90–94_=22, *N*_95–99_=21	*N*_90–94_=105, *N*_95–99_=92	
Lung	**1.39**	**0.81**	*χ*^2^_1_=3.82 (*P*=0.051)
	(0.81, 2.38)	(0.72, 0.91)	
	*N*_90–94_=23, *N*_95–99_=32	*N*_90–94_=656, *N*_95–99_=531	
Prostate	**1.71**	**0.93**	*χ*^2^_1_=3.27 (*P*=0.071)
	(0.89, 3.31)	(0.82, 1.07)	
	*N*_90–94_=14, *N*_95–99_=24	*N*_90–94_=443, *N*_95–99_=413	
			
**Females**			
All cancers	**1.24**	**0.91**	*χ*^2^_1_=10.0 (*P*=0.0015)
	(1.03, 1.49)	(0.86, 0.96)	
	*N*_90–94_=206, *N*_95–99_=255	*N*_90–94_=3000, *N*_95–99_=2731	
Breast	**1.37**	**0.81**	*χ*^2^_1_=7.70 (*P*=0.0055)
	(0.96, 1.97)	(0.73, 0.90)	
	*N*_90–94_=70, *N*_95–99_=51	*N*_90–94_=816, *N*_95–99_=661	
Colorectal	**2.33**	**0.95**	*χ*^2^_1_=5.37 (*P*=0.020)
	(1.07, 5.09)	(0.83, 1.09)	
	*N*_90–94_=9, *N*_95–99_=21	*N*_90–94_=398, *N*_95–99_=378	
Head and neck	**1.33**	**1.33**	*χ*^2^_1_=0.001 (P=0.99)
	(0.72, 2.46)	(0.93, 1.90)	
	*N*_90–94_=18, *N*_95–99_=24	*N*_90–94_=52, *N*_95–99_=69	
Lung	**1.50**	**1.11**	*χ*^2^_1_=0.43 (P=0.51)
	(0.61, 3.67)	(0.94, 1.30)	
	*N*_90–94_=8, *N*_95–99_=12	*N*_90–94_=304, *N*_95–99_=333	

Number of cancer cases for 1990–1994: *N*_90–94_; Number of cancer cases for 1995–1999 *N*_95–99_. Person-years assumed same for 1990–1999.

). Interactions with deprivation were also explored.

## RESULTS

Out of 12 128 cancer cases identified, 862 were classified as occurring in South Asians (7%). South Asians (median age 61.5 years) were younger than non-South Asians (median age 71.9 years).

Overall cancer rates adjusted for age and deprivation differences were lower for South Asians than non-South Asians (males: IRR=0.61 95% confidence interval (CI) (0.55, 0.68); females IRR=0.75 (0.68, 0.82)). However, this pattern changed significantly with age (Table 1). Although older South Asians had much lower rates of cancer than the rest of the population, younger South Asians were at increased risk compared with non-South Asians.

South Asians had significantly lower rates of colorectal, lung, breast and prostate cancer, but a higher rate of head and neck cancer. For colorectal cancer, trends with age were similar to all cancers combined, with increasing IRRs among younger age groups, although for women this was not formally significant. For men, head and neck cancer showed the opposite pattern, with a decrease in risk among younger South Asians. There was little evidence of a pattern with age for lung, prostate, breast or female head and neck cancer.

Time trends for all cancers combined (Table 2) were different for South Asians compared with the rest of the population. Adjusted incidence rates increased over time for South Asians (men: +28%; women: +24%), but decreased for non-South Asians (men: −5%; women: −9%). For men, time trends differed by deprivation tertile. South Asians from the most deprived tertile had much higher increases in cancer risk (IRR=1.93 (1.41, 2.65)) than those from the least deprived tertile (IRR=1.08 (0.83, 1.39)). For non-South Asian men, the time trend with deprivation was less steep with those from the most deprived tertile having little change in risk (IRR=1.02 (0.93, 1.11)) while those from the least deprived tertile had a decrease in risk (IRR=0.92 (0.84, 1.01)). For women, changes over time were similar for all deprivation tertiles.

For individual cancer sites, time trends were different for men and women. For men, lung and prostate cancer were seen to be increasing among South Asians and decreasing among the rest of the population. For lung cancer, this pattern changed with deprivation tertile among non-South Asians, with those from the least deprived tertile having little change in incidence over time, while those from the most deprived tertile showed a decrease in incidence. The incidence of colorectal and head and neck cancer showed little change over time for all men.

South Asian women showed increasing rates of colorectal and breast cancer over time, while the rates decreased among the rest of the population. Lung and head and neck cancer incidence increased over time for all women with no differences by ethnicity.

## DISCUSSION

These findings in Leicester confirm that overall South Asians have lower rates of cancer than the rest of the population (Winter *et al*, 1999). However, they show that this is changing with age and time. While older South Asians have a much lower risk of cancer, those from younger age groups, particularly children, have a higher risk. Most importantly, over the last 10 years, cancer rates have increased among South Asians while they have fallen among the rest of the population.

Incidence of head and neck cancers among South Asians often receives attention because these cancers are *relatively* more common among South Asians than the rest of the population. Here we have shown that for men this pattern is changing with rates among younger age groups approaching those of the rest of the population. Furthermore, Bhopal and Rankin (1996) have highlighted the need to look at *absolute* numbers of cancer cases. In Leicester, lung cancer is the most common cancer for both South Asian and non-South Asian men, and numbers of colorectal, breast and prostate cancer among South Asians are rapidly increasing.

Cancer incidence appears to be increasing quite rapidly among South Asians. This suggests that health promotion programmes should target the whole population and that any tendency to regard South Asians as ‘low-risk’ populations for cancer needs to be rethought.
